# Effect of pipe material and disinfectant on active bacterial communities in drinking water and biofilms

**DOI:** 10.1093/jambio/lxaf004

**Published:** 2025-01-06

**Authors:** Sallamaari Siponen, Jenni Ikonen, Vicente Gomez-Alvarez, Anna-Maria Hokajärvi, Matti Ruokolainen, Balamuralikrishna Jayaprakash, Mikko Kolehmainen, Ilkka T. Miettinen, Tarja Pitkänen, Eila Torvinen

**Affiliations:** 1 Department of Environmental and Biological Sciences, University of Eastern Finland, P.O. Box 1627, FI-70211 Kuopio, Finland; 2 Department of Public Health, Finnish Institute for Health and Welfare, P.O. Box 95, FI-70701 Kuopio, Finland; 3 Office of Research and Development, U.S. Environmental Protection Agency, 26 W. Martin Luther King Dr., Cincinnati, OH 45268, United States; 4 Water and Environmental Engineering, Aalto University, Tietotie 1E, FI-02150 Espoo, Finland; 5 Faculty of Veterinary Medicine, Department of Food Hygiene and Environmental Health, University of Helsinki, P.O. Box 66, FI-00014 Helsinki, Finland; 6 Present Address: City of Kuopio, Environmental Health Services, P.O. Box 228, FI-70101 Kuopio, Finland

**Keywords:** chlorine, chloramine, plastic pipes, copper pipes, opportunistic pathogens

## Abstract

**Aims:**

We investigated the combined effects of pipe materials and disinfection chemicals on bacterial community and its active RNA fraction in water and biofilms in a pilot-scale premise plumbing system.

**Methods and results:**

The changes in bacterial communities were studied within four pipelines using copper and cross-linked polyethylene (PEX) pipe with chlorine or chloramine disinfection. The total and active bacterial communities and the presence of opportunistic pathogens (*Legionella* spp. and *Mycobacterium* spp.) were analyzed using 16S rRNA (gene) amplicon sequencing. The dominant classes were *Alphaproteobacteria* (31%) and Gammaproteobacteria (24%). Class Planctomycetia was increased in active fraction of chlorinated waters and PEX pipe biofilms and decreased in chloraminated waters and copper pipe biofilms. The alpha diversity of the active fractions in biofilms were highest in chloraminated PEX pipe samples (Chao1 mean = 163, *P* < 0.05, Kruskal–Wallis). *Legionella* spp. was more abundant and active in waters treated with chlorine than chloramine.

**Conclusions:**

Disinfectant had a stronger impact than pipe material on the bacterial community composition in water. A combined effect of pipe material and disinfectant was more evident on the composition and activity of the biofilm communities than the individual effect of copper, PEX, chlorine, or chloramine.

## Introduction

Microbial quality of drinking water changes in drinking water distribution systems (DWDSs) and premise plumbing may deteriorate the water quality if the system is not managed properly and circumstances favor the growth of microbes (Ji et al. 2015, Douterelo et al. 2019, LeChevallier et al. 2024). In drinking water pipelines, microbes inhabit the inner surfaces of pipes, forming biofilms, and the detaching bacteria may again act as a source of bacteria to water (Fish et al. 2017, Goraj et al. 2021, Learbuch et al. 2022, Erdei-Tombor et al. 2024). The formation and microbial composition of the biofilms mainly depend on the microbiological and chemical quality of the distributed drinking water and on the circumstances, such as temperature and hydraulic conditions, prevailing in the distribution system (Lehtola et al. 2004, Ji et al. 2015, Liu et al. 2017, Douterelo et al. 2019, Cowle et al. 2020). Biofilm consisting of bacteria cells, other microbes, and extracellular polymeric substances offers protection for bacteria against antimicrobial agents and provides physicochemical stability (Fish et al. 2017, Santos et al. 2018, Douterelo et al. 2019). Several studies have shown that biofilms in pipelines inside buildings can represent reservoirs that also support the growth of opportunistic pathogens such as *Legionella* and *Mycobacterium* (Cullom et al. 2020, Falkinham 2020).

To maintain a residual concentration of disinfection long after the application point, the most used disinfectants in drinking water treatment are chlorine and chloramine (Dias et al. 2019, Ricca et al. 2019). Disinfection using chlorine compounds affects microbial community structure (Dias et al. 2019, Inkinen et al. 2021, Siponen et al. 2024) and their functional genes (Tiwari et al. 2022, Gomez-Alvarez et al. 2023) in water and biofilms in DWDSs. Chlorine effectively decreases bacterial activity and diversity and is effective against opportunistic pathogens (Buse et al. 2019, Potgieter et al. 2021, Kim et al. 2024). However, it is not as stable a chlorine compound as chloramine, which also forms less regulated disinfection by-products (Liu et al. 2016, Ding et al. 2019). Chloramine is commonly used in large DWDSs to maintain disinfection chemical residue within the whole network (Allard et al. 2020, Oliveira et al. 2024). Chloramine is also effective against opportunistic pathogens, such as *Legionella*, although these opportunistic pathogens originating from natural waters and soil are challenging to entirely eradicate from DWDSs (Lytle et al. 2021, Kim et al. 2024).

Composition of bacterial community structures and existence of opportunistic pathogens are affected by pipe material (Douterelo et al. 2020, Goraj et al. 2021, Tang et al. 2021). Metals, such as copper and iron, and plastics, such as polyvinyl chloride and cross-linked polyethylene (PEX), are common pipe materials in water pipes in premise plumbing systems (Cullom et al. 2020). Nutrients leaching from plastic pipes may enhance bacterial growth in pipes but not at a similar magnitude in all plastic pipe materials (Neu and Hammes 2020). Copper pipes have been shown to control biofilm formation at first in new pipes (Lehtola et al. 2004, Gomes et al. 2019). Furthermore, when comparing bacterial communities in biofilms, lower amounts of mycobacteria have been reported from copper pipes than plastic pipes (Lu et al. 2014, Inkinen et al. 2018). Some chemical properties of water, including pH, phosphate concentration, and natural organic matter, may, however, prevent antimicrobial effects of copper (Song et al. 2021). Pipe material also affects the effectiveness of disinfection chemicals (Mutoti et al. 2007, Tolofari et al. 2020). Copper corrosion byproducts may enhance chlorine decay (Lytle and Liggett 2016, Ding et al. 2019). When disinfectants and pipe materials have been investigated together, free chlorine has observed to be more effective disinfectant against plastic pipe biofilms than chloramination but in contrast, chloramine has been more effective on other pipe materials including copper (Buse et al. 2019, Li et al. 2020).

To elucidate the ecology of microbes and the presence of opportunistic pathogens in DWDSs, many studies have used 16S rRNA gene-based methods to analyze bacterial communities (Ji et al. 2015, Cowle et al. 2020, Lee et al. 2021). However, this DNA-based method does not provide information on whether bacteria are dead or alive (Li et al. 2017). It is important to study the activity of bacteria, as living active bacteria may effectively deteriorate water quality and cause infections, unlike dead bacteria cells. Ribosomal RNA is actively produced and regulated by living bacteria cells and it degrades more quickly than DNA after cell death (Li et al. 2017). Therefore, RNA-based methods can be used to evaluate active and dormant bacteria (Pitkänen et al. 2013, Li et al. 2017), but only limited number of studies concerning bacterial communities in drinking water has been published (Inkinen et al. 2018, Siponen et al. 2024).

The composition of active and dormant members of bacterial communities in drinking water networks remains unclear. Consequently, our objective was to assess the effect of copper and plastic pipe combined with chlorine and chloramine disinfection to determine how these combinations affect active fraction of bacterial community in comparison to total community at early phases of biofilm formation. A further objective was to examine the opportunistic pathogens *Legionella* and *Mycobacterium* in bacterial communities to determine pipe material and disinfection chemical-related health risks and controlling opportunities.

## Materials and methods

### Experimental set-up

**Figure 1.**
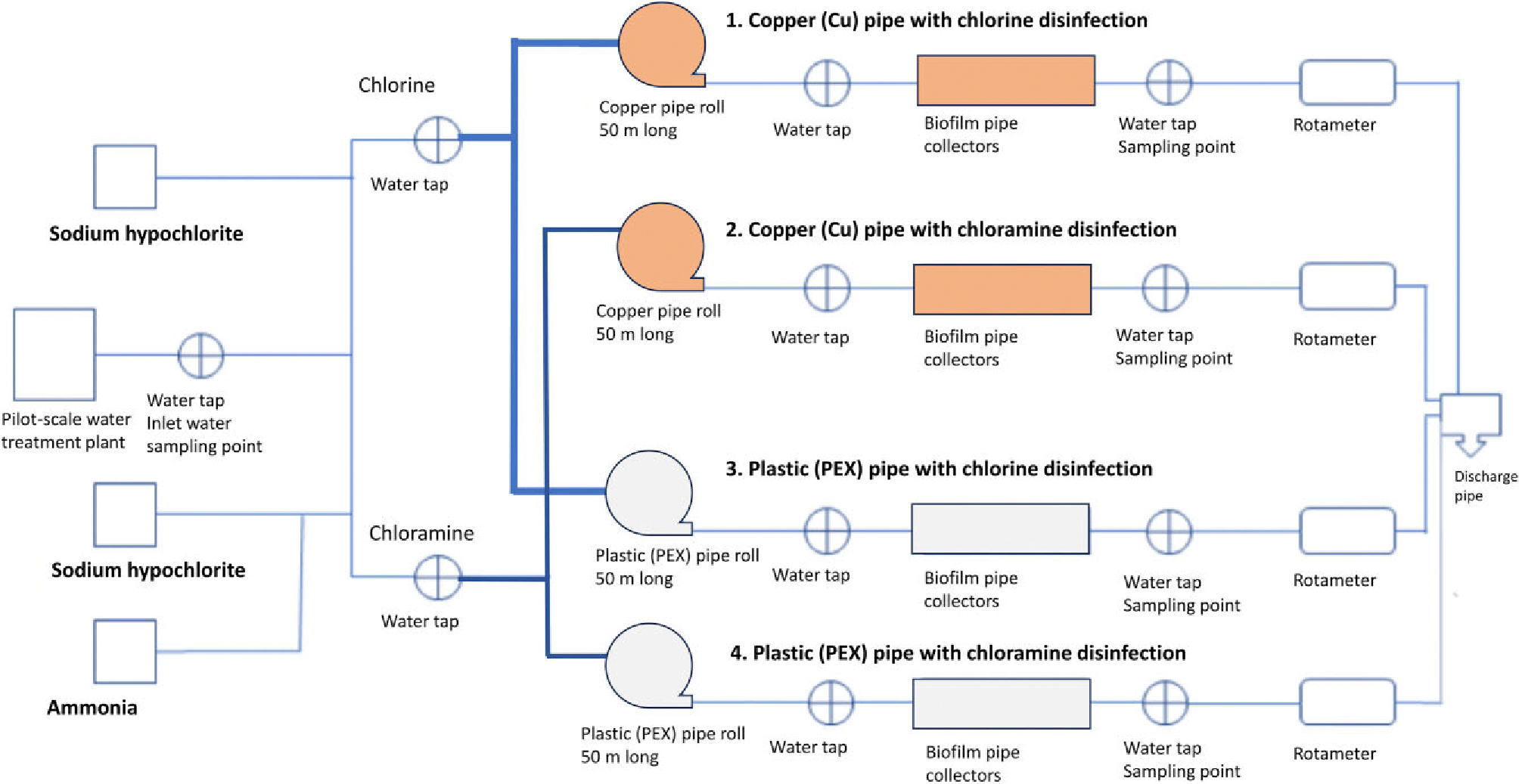
Pilot-scale DWDS with four lines, two of copper and two of cross-linked polyethylene (PEX), built for the study. Hypochlorite and chloramine disinfection systems, water sampling points, and biofilm collectors are shown.

Bacterial community structure in a pilot-scale DWDS described by Brester et al. (2020) was investigated. DWDS consisted of four pipelines: two of copper and two of plastic, more precisely PEX, with sodium hypochlorite or chloramine disinfection (Fig. 1). Pipelines with inner diameter of 10 mm consisted of 50 m long pipe rolls (Fig. 1) and 38 biofilm collectors (each 0.15 m in length) in the beginning of the experiment. Water flow was constant and set to 250 ml min^−1^, 0.053 m s^−1^, and was laminar with a calculated Reynolds number of 525. Two stagnations, 2-h and 6-h stagnations, were between sampling weeks 11 and 12 due to maintenance of the water treatment plant.

The water distribution system was operated for a total of 19 weeks, from the beginning of June until mid-October as described earlier by Brester et al. (2020). The system was operated without disinfection for 9 weeks, after which disinfection was applied for 10 weeks (Table S1). A median total chlorine concentration of water flowing to the pipelines was 0.5 mg l^−1^. Water for the system was supplied by a pilot-scale drinking water treatment plant using surface water from the nearby lake and described earlier by Hokajärvi et al. (2018). Treatment included coagulation, flotation, sand filtration, and alkalinization. From the pilot-scale water treatment plant, water flowed through the 20 m PEX pipe before arriving to the location where it was first divided into two lines for two different disinfection methods and then divided into two different pipe material lines, thus comprising a total of four study lines.

### Water and biofilm sampling

The biofilms of the system were formed in copper and PEX pipes by letting water flow through the pipes (at rate 250 ml min^−1^) for 3 weeks (20 days) before the first sampling (sampling week 1, Table S1). Sampling was continued weekly for 7 weeks (sampling weeks 1–7) before starting disinfection with two different chlorine compounds. At sampling week 7, samples were collected a day before the start of disinfection (7a) and a day after start of disinfection (7b). Samples during the disinfection were collected for 11 consecutive weeks (sampling weeks 7–17). In the weekly sampling, a biofilm sample and water sample were collected from each of the four pipelines. Once a month an inlet water sample of water coming from the pilot-scale water treatment plant was taken to determine the inlet water quality without the effect of disinfection or pipe material. Large-volume samples (100 l) of inlet water and study pipelines were collected once in the last week of the study. Physicochemical analyses and determination of heterotrophic plate count (HPC) from inlet water and waters and biofilms from the four pipelines were conducted weekly (Table S1).

Water samples for microbiological analyses were collected in 3 × 1 l sterile plastic bottles. Bottles contained sodium thiosulfate, and 50 μl of sodium thiosulfate solution (18 mg l^−1^) was also added to each piece of biofilm pipe collectors. Biofilm pipe collectors made of copper and PEX were made of 15 cm pieces with an inside diameter of 10 mm. Biofilm from the inside of two pipe collectors from each pipeline was removed as described by Inkinen et al. (2019) by shaking 22.5 Hz for 3 × 5 min (Heidolph Vibramax, Schwabach, Germany) with sterile 2 mm glass beads (Karl Hecht GmbH & Co. KG, Sondheim, Germany) followed by rinsing with a 5 ml sample water from the same sample point. The volumes of biofilm samples were 34–39 ml. Large-volume water samples (100 l) were concentrated using dead-end ultrafiltration (DEUF) method as earlier described by Inkinen et al. (2019).

### Physicochemical parameters

Turbidity (NTU) was measured spectrophotometrically at a wavelength of 860 nm with a Turb 555IR spectrophotometer (WTW GmbH & Co. KG, Weilheim, Germany). Absorbance and UV-absorbance were assayed at wavelengths of 420 and 254 nm, respectively (Shimadzu UV-1601, Shimadzu Co., Kyoto, Japan). pH and electric conductivity (EC) were assayed using a Multi 3430i meter (WTW GmbH & Co. KG, Weilheim, Germany). Total chlorine, free ammonia, and nitrite were determined by using Hach Lange DR 2800 spectrophotometer (Hach Lange GmbH, Düsseldorf, Germany, methods 8167 for total chlorine, 10 200 for free ammonia, and 8507 for nitrite) according to the manufacturer’s instructions. Metal analyses, including the measurements of copper and iron, were determined by using a Hach Lange DR2800 spectrophotometer (Hach Lange GmbH, Düsseldorf, Germany, methods 8506 for Cu and 8008 for Fe). Microbially available phosphorus (MAP), acetate carbon, and assimilable organic carbon (AOC) were analyzed as described by Ikonen et al. (2017). All physicochemical parameters were measured from water samples. Copper and iron concentrations were also measured from biofilm samples.

### Microbiological parameters

Microbiological parameters were measured from water and biofilm samples. HPC was used to enumerate heterotrophic bacteria, yeasts, and molds, as described by Ikonen et al. (2017). Samples were inoculated on a Reasoner’s 2 Agar (R2A) medium (Difco, Detroit, MI, USA) and incubated at 22°C ± 2°C for 7 days. Total microbial cell counts were preserved by adding 0.22 *μ*m filtered 37% formaldehyde to the sample to reach a final concentration of 2%, stained with DAPI (4.6-diamidino-2-phenylindole dihydrochloride) (Merck, Darmstadt, Germany), and visualized with an Olympus BX51TF epifluorescence microscope (Olympus Co., Japan). High-sensitivity luminometer Lumitester C-110 (Kikkoman, Japan) with ATP Biomass Kit HS (Bio-Thema, Sweden) was used for measuring adenosine triphosphate (ATP) concentrations.

### Nucleic acid extraction and amplicon sequencing

Water samples (1 l), biofilm samples (27–32 ml), and DEUF concentrates (100 ml corresponding to 17.4–18.2 l of original water) were filtered on polyethersulfone (PES) membrane filters with pore size of 0.22 *μ*m (Express Plus Membrane, Merck Millipore, Ireland), after which the filters were stored at −75°C or lower. Total nucleic acids were extracted as previously described by Inkinen et al. (2019) and Brester et al. (2020). In brief, Chemagic DNA Plant Kit (Perkin Elmer, Waltham, MA, USA) was used, and RNA was further purified using Ambion Turbo DNA-free DNase kit (Life Technologies, Carlsbad, CA, USA). cDNA was synthesized with the Invitrogen Superscript IV VILO system (Thermo Fisher Scientific, Waltham, MA, USA) and used in the 16S rRNA analysis.

Active and dormant and total bacterial communities were studied using amplicon sequencing for 16S ribosomal RNA (rRNA, further in text named as active fraction) and rRNA gene (rDNA, further in text named as total fraction). The nucleic acids were used as templates for polymerase chain reaction amplification with the modified primer sets 341F (5′-CCTACGGGNGGCWGCAG-3′) and 785R (5′-GACTACHV GGGTATCTAAKCC-3′) (Herlemann et al. 2011, Klindworth et al. 2013). Sequencing was done on an Illumina MiSeq using V3 Chemistry (LGC Genomics GmbH, Berlin, Germany) as previously described by Inkinen et al. (2019).

### Sequence data processing and statistical analyses

Data were denoised by using the DADA2 protocol (software version 1.8) to produce amplicon sequence variants (ASVs, Callahan et al. 2016). The sequence table was constructed, and chimeras were removed using a “per-sample” method (Callahan et al. 2016). Taxonomy of sequences was obtained using database GTDB R207 (released in April 2022). Also, the taxonomic nomenclature used here is from the database GTDB R207. Sequence processing of the samples included negative and positive controls. Sequence counts and alpha and beta diversity of samples were compared to DNA and RNA negative and positive controls to check the quality of samples and to set a limit for exclusion of samples with too low sequence count. One ASV was abundant in all controls and samples and was identified as a contaminant from the nucleic acid extraction step. This ASV 08378 was removed from the data. Also, ASV 00002, ASV 00229, ASV 01073, ASV 01094, ASV 01462, ASV 01640, ASV 01687, and ASV 01930 occurred unexpectedly in negative controls but not at all or only in low numbers in samples and were removed from the data. Active RNA fractions of samples from sampling weeks 1–10 had to be excluded from further analysis as they did not pass the quality control. Further, all samples with under 1009 sequence reads were excluded from analysis. Thus, total DNA fractions of biofilm samples with low sequence counts, especially in samples from disinfected copper lines, were therefore excluded from analysis. For further bacterial community composition analysis, there were five sample groups of bacterial communities: (i) total (DNA) bacterial communities of water samples before and (ii) during disinfection, (iii) water and (iv) biofilm samples of active (RNA) bacterial communities during disinfection in the last seven weeks, and (v) total (DNA) and active (RNA) communities of large-volume water samples in the last week of the study (Table 1).

Sequence data of samples were rarefied to the smallest sequence count of the sample group (above 1008) in MicrobiomeAnalyst. For statistical analysis and for drawing figures, MicrobiomeAnalyst and IBM SPSS Statistics software were used. Alpha and beta diversity and taxonomy of bacteria were analyzed using MicrobiomeAnalyst. Alpha diversity index Chao1, physicochemical parameters, bacteria count, and abundance of *Legionella* spp. and *Mycobacterium* spp. were compared between different sample groups and tested if the difference was significant with non-parametric Kruskal–Wallis test in IBM SPSS (version 29). Beta diversity between sample groups was analyzed using Bray–Curtis dissimilarity index. The permutation-based analysis of variance (PERMANOVA) method was used in MicrobiomeAnalyst to calculate *R*^2^, which shows the proportion of the variance from 0 to 1 explained by the groups. *R*^2^ = 1 indicates that communities of different tested sample groups are completely dissimilar. Weekly bacteria class changes were calculated, and figures produced in Microsoft Excel. Bacteria content changes of inlet water over time were considered when comparing weekly changes in water and biofilm samples by subtracting inlet water bacteria (%) from bacteria in water and biofilm samples (%). Percentage point change of total (DNA) fractions for weeks 1–4 was calculated by subtracting percentages of inlet water of week 1, for weeks 5–10 by subtracting inlet water of week 5, and for weeks 11–16 by subtracting inlet water of week 16. Percentage point change of active (RNA) fractions for weeks 11–14 was calculated by subtracting percentages of inlet water of week 12 and for weeks 15–17 by subtracting inlet water of week 16.

## Results

In total, bacterial communities of 107 samples were analyzed. Our study generated 1 365 403 sequences and 5367 ASVs were identified after libraries with <1009 sequences were removed from analysis. The maximum sequence count per sample was 77 877.

### Diversity and taxonomy of bacteria communities

**Figure 2.**
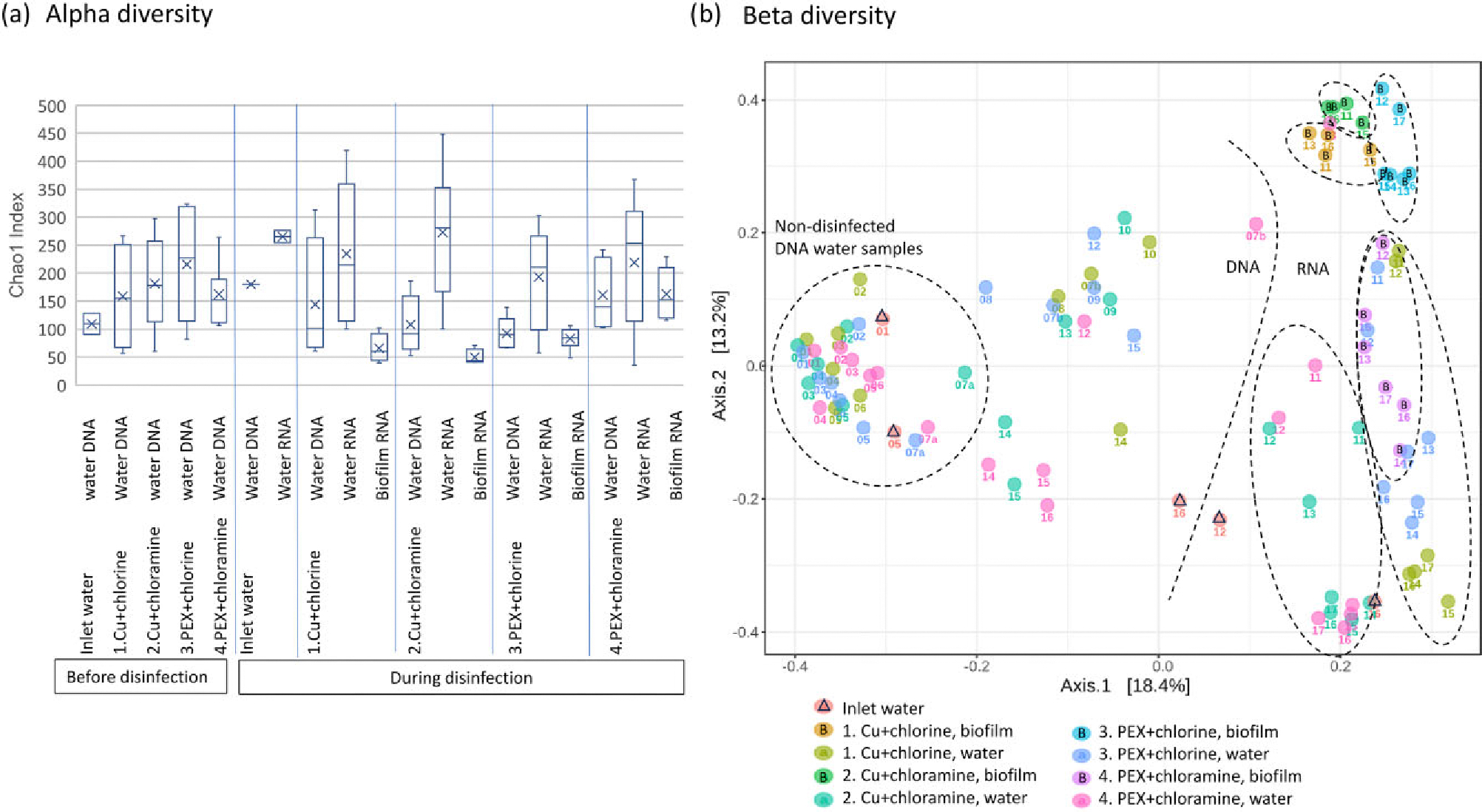
Alpha diversity (a) and beta diversity (b) of water and biofilm samples of total DNA fraction before and during disinfection at sampling weeks 1–17 and active RNA fraction during disinfection at weeks 11–17. Alpha diversity is calculated by Chao1 index and box plot shows upper and lower quartiles, median value as line, mean value as X, and outliers as circles. Beta diversity is shown by principal coordinate analysis (PCoA) plot and calculated by Bray–Curtis dissimilarity index. Indicated below each sample is the week numbers that a sample was collected. Disinfection began in week 7 (samples taken on week 7 before disinfection marked as 7a). B = biofilm, Cu = copper pipe, and PEX = cross-linked polyethylene pipe.

In the total fraction of bacterial community, species richness only decreased in water obtained from chlorinated PEX pipeline (line 3), as Chao1 index was significantly higher (*P* < 0.05, Kruskal–Wallis test) in water before disinfection (mean = 220, *n* = 7) compared to disinfected samples (mean = 93, *n* = 5) (Fig. 2a). The alpha diversity in total fraction of community of all water samples increased during weeks 1–7, from a mean value of 110 at week 1 (*n* = 4) to a mean value of 170 at week 7 (*n* = 3). The alpha diversity decreased to 110 (*n* = 3) after the disinfection was started at week 7 (Fig. S1).

In active fraction of communities of disinfected water samples, species richness did not significantly differ between the four pipelines (*P* > 0.27, Kruskal–Wallis test, Fig. 2a). The species richness (Chao1 index) of active fraction of water samples of the four lines in total increased from 100 (*n* = 3) at week 13–340 (*n* = 4) at week 14 and maintained a similar richness until the end of the study (Fig. S1). A moderate increase in alpha diversity was observed for biofilms during weeks 14–17. Chao1 index in disinfected RNA biofilm samples was higher in chloraminated PEX pipe (mean = 160, *n* = 6, *P* < 0.05, Kruskal–Wallis test) than in the chlorinated PEX pipe (mean = 83, *n* = 6) or in chlorinated and chloraminated copper pipes (mean = 66, *n* = 4; mean = 50, *n* = 4, respectively) (Fig. 2a). Alpha diversity was lower in disinfected biofilms than in inlet water (mean = 270, *n* = 2).

Total fraction of bacterial communities of water samples and active fraction of communities of water and biofilm samples (Fig. 2b) yielded different community composition in beta diversity analysis using Bray–Curtis dissimilarity index (*R*^2^: 0.16; *P* = 0.001, *n* = 96, PERMANOVA). Also, water samples of total fraction before disinfection formed separated cluster from disinfected samples, showing dissimilarity between bacterial communities (*R*^2^: 0.16; *P* = 0.001, *n* = 45, PERMANOVA). Dissimilarity between bacterial communities was observed also in inlet water samples before and during disinfection (Fig. 2b). In disinfected water samples of total fraction, the difference between disinfection chemical explained only slightly the dissimilarity between community compositions of samples (*R*^2^: 0.10, *P* < 0.05, *n* = 18, PERMANOVA), whereas pipe material did not significantly (*P* = 0.46, *n* = 18, PERMANOVA) explain the dissimilarity between samples (Fig. 2b).

Active fractions of bacterial communities showed dissimilarity between four pipelines during disinfection in beta diversity analysis (*R*^2^: 0.26; *P* = 0.001, *n* = 27, PERMANOVA). Active fractions of chlorinated water samples of both pipe materials were clustered separately (Fig. 2b) showing dissimilarity compared to chloraminated water samples of both pipe materials (*R*^2^: 0.19; *P* = 0.001, *n* = 27, PERMANOVA). Beta diversities of bacterial communities of chloraminated water samples were more similar to inlet water samples than chlorinated water samples. Disinfected biofilm samples of active fraction contained different bacterial community structures than waters, except for the PEX pipeline with chloramine, where biofilm samples clustered close to chlorinated water samples (Fig. 2b). In biofilms, each pipeline separated into its own clusters showing dissimilarity in bacterial communities between pipelines (*R*^2^: 0.44; *P* = 0.001, *n* = 20, PERMANOVA). Biofilms from copper pipes disinfected with chlorine and chloramine (lines 1 and 2, Fig. 2b) are clustered close to each other and close to the cluster of chlorinated PEX pipe biofilm (line 3) showing more similarity between these pipe biofilms compared to biofilm samples from chloraminated PEX pipes (line 4) that clustered together with chlorinated water samples (Fig. 2b).

**Figure 3.**
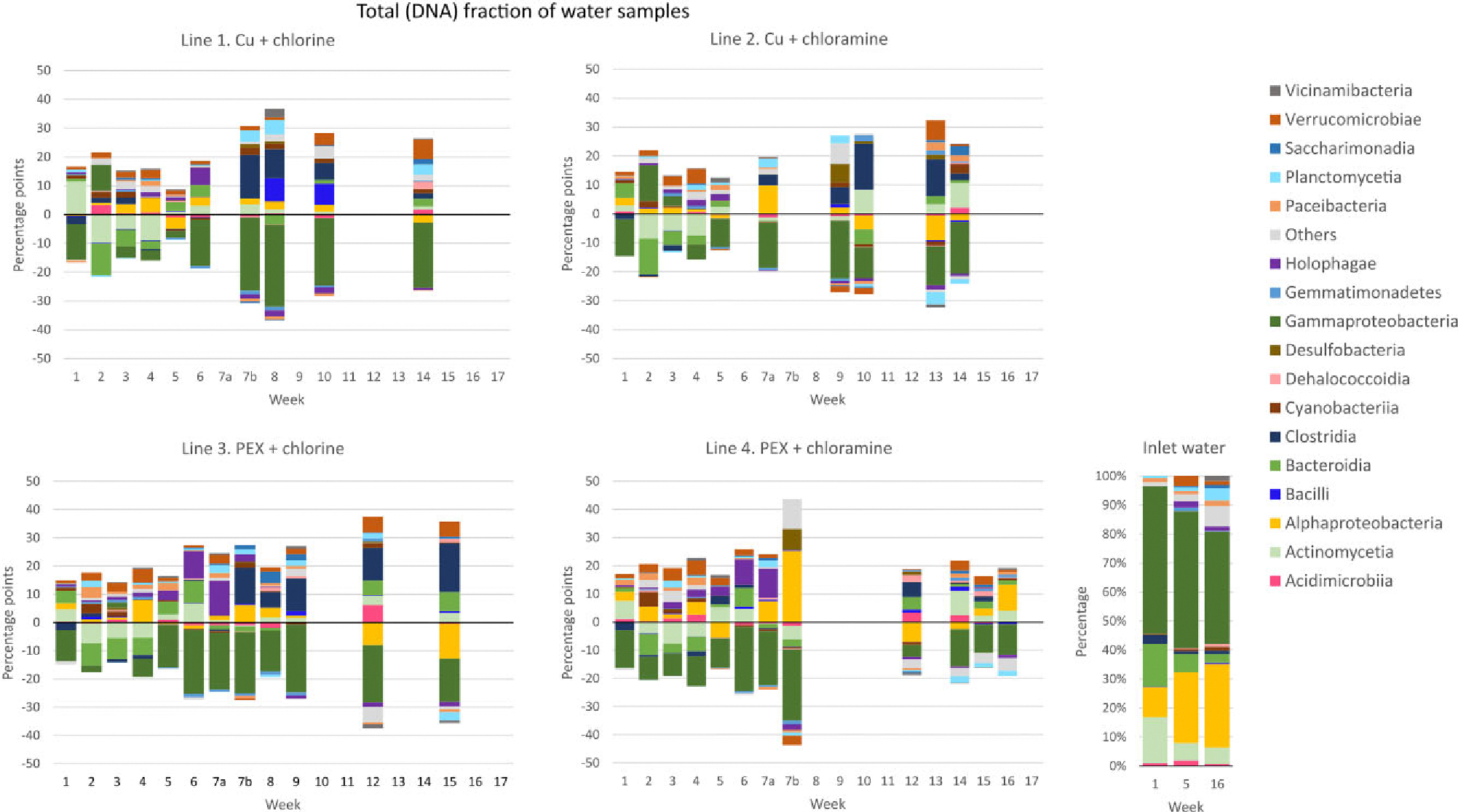
Difference in relative abundance of bacterial classes measured from total DNA fraction of the water samples in lines 1–4, as compared with relative abundance of bacterial classes in the inlet water in sampling weeks 1–7a before disinfection and 7b–17 during disinfection. For some weeks, the taxonomy data are missing due to low sequence count (below 1009) in the samples. The group “Others” contains classes with sequence count <200. Percentage point change for weeks 1–4 was calculated by subtracting percentages of inlet water of week 1, for weeks 5–10 by subtracting inlet water of week 5, and for weeks 11–16 by subtracting inlet water of week 16. Cu = copper pipe and PEX = cross-linked polyethylene pipe.

**Figure 4.**
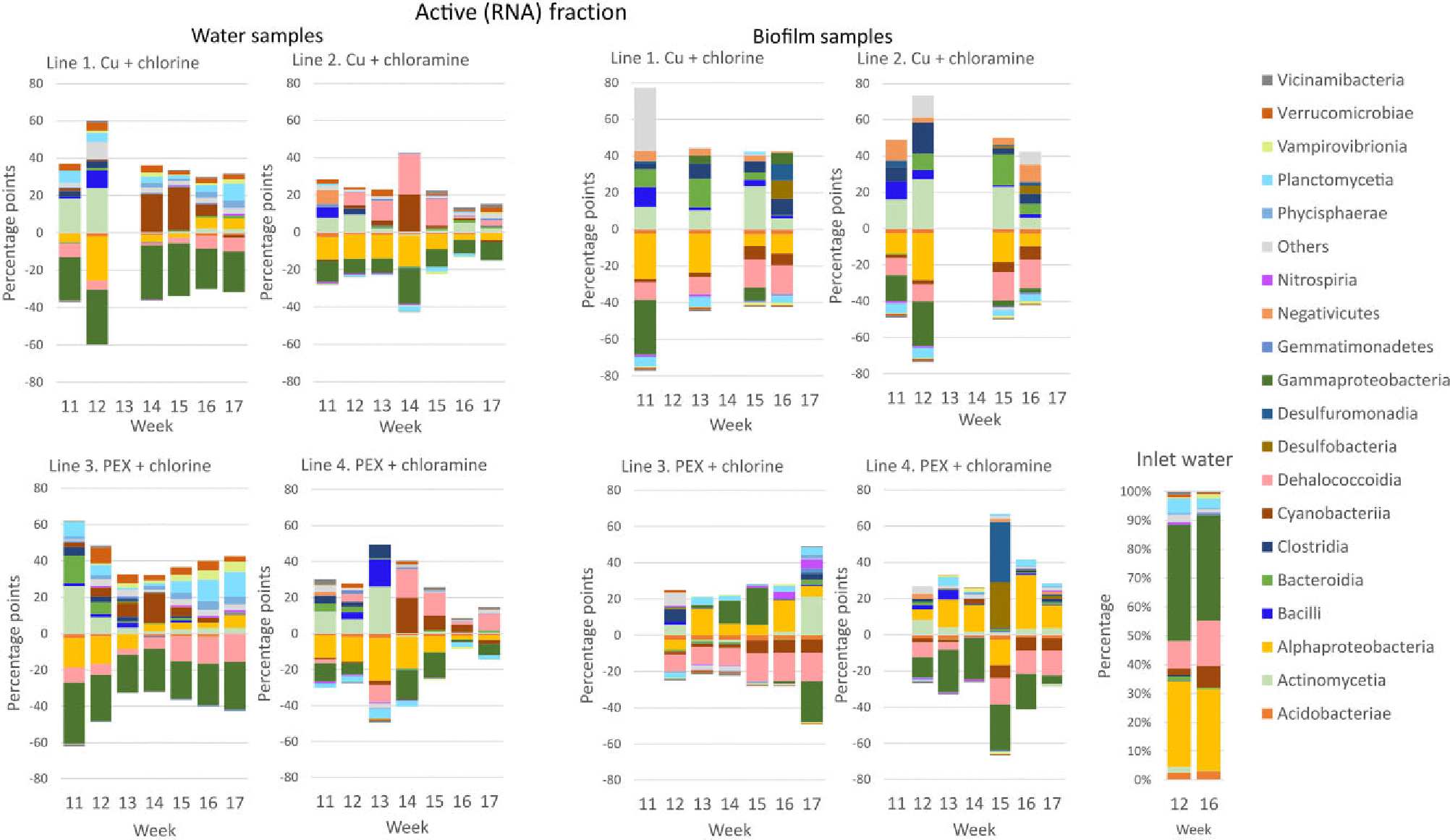
Difference between the relative abundance of bacterial classes in active RNA fractions on water and biofilm samples in lines 1–4 as compared with bacterial classes in the inlet water in sampling weeks 11–17 during the disinfection. For some weeks, the taxonomy data are missing due to low sequence count (below 1009) in the samples. Group “Others” contains classes with sequence count <200. Percentage point change for weeks 11–14 was calculated by subtracting percentages of inlet water of week 12 and for weeks 15–17 by subtracting inlet water of week 16. Cu = copper pipe and PEX = cross-linked polyethylene pipe.

Overall, taxonomy profile showed that *Alphaproteobacteria* (31%) and Gammaproteobacteria (24%) were the most abundant classes, followed by Actinomycetia (8%), Bacteroidia (5%), Dehalococcoidia (4%), Planctomycetia (4%), and Cyanobacteriia (4%). *Alphaproteobacteria* and Gammaproteobacteria were the dominant bacteria classes in inlet water in both active and total fraction. In total fraction of inlet water (Fig. 3), the relative abundances of Actinomycetia (6%–16%) and Bacteroidia (3%–15%) were higher than in active fraction (both classes ≤2%, Fig. 4), whereas in active fraction abundances of Dehalococcoidia (10%–16%) and Cyanobacteriia (2–7%) were higher (Fig. 4) than in total fraction (both classes <1%, Fig. 3). In total fraction of water samples, the abundance of *Alphaproteobacteria* increased in all four study lines, whereas the abundance of Gammaproteobacteria decreased relative to inlet water during the weeks before the addition of disinfectants (weeks 1–7a in Fig. 3). Similar, the abundance of Holophagae increased during the initial weeks but the change was noticeable clearer in water samples taken from the PEX pipe than from copper pipes. After the start of disinfection, the relative abundance of Clostridia increased and Holophagae slightly decreased (weeks 7b–17 in Fig. 3).

In active fraction, the abundance of *Alphaproteobacteria*in chlorinated water decreased in the first weeks and then increased, whereas in chloraminated water *Alphaproteobacteria* decreased in all weeks compared with inlet water (Fig. 4). Like total fraction, *Gammaproteobacteria* decreased with both disinfection chemicals but more in chlorinated waters. The increase in abundances of *Planctomycetia*, *Verrucomicrobiae*, *Vampirovibrionia*, and *Phycisphaerae* was higher in chlorinated waters than in chloraminated waters and was highest in the chlorinated PEX pipeline. The abundance of *Dehalococcoidia* increased in chloraminated waters and decreased in chlorinated waters in active fraction. Classes *Cyanobacteria* and *Actinomycetia* mainly increased or stayed at the same level in all four study lines. In biofilms, *Gammaproteobacteria* did not decrease as strongly as in water samples, except in chloraminated PEX pipeline 4 (Fig. 4). In copper pipe biofilms, a higher increase in *Actinomycetia*, *Bacteroidia*, *Clostridia*, and *Negativicutes* was observed than in PEX pipes. In PEX pipe biofilms, *Alphaproteobacteria* and *Planctomycetia* increased, whereas in copper pipes their abundance decreased. *Dehalococcoidia* and *Cyanobacteria* decreased in all pipeline biofilms.

**Figure 5.**
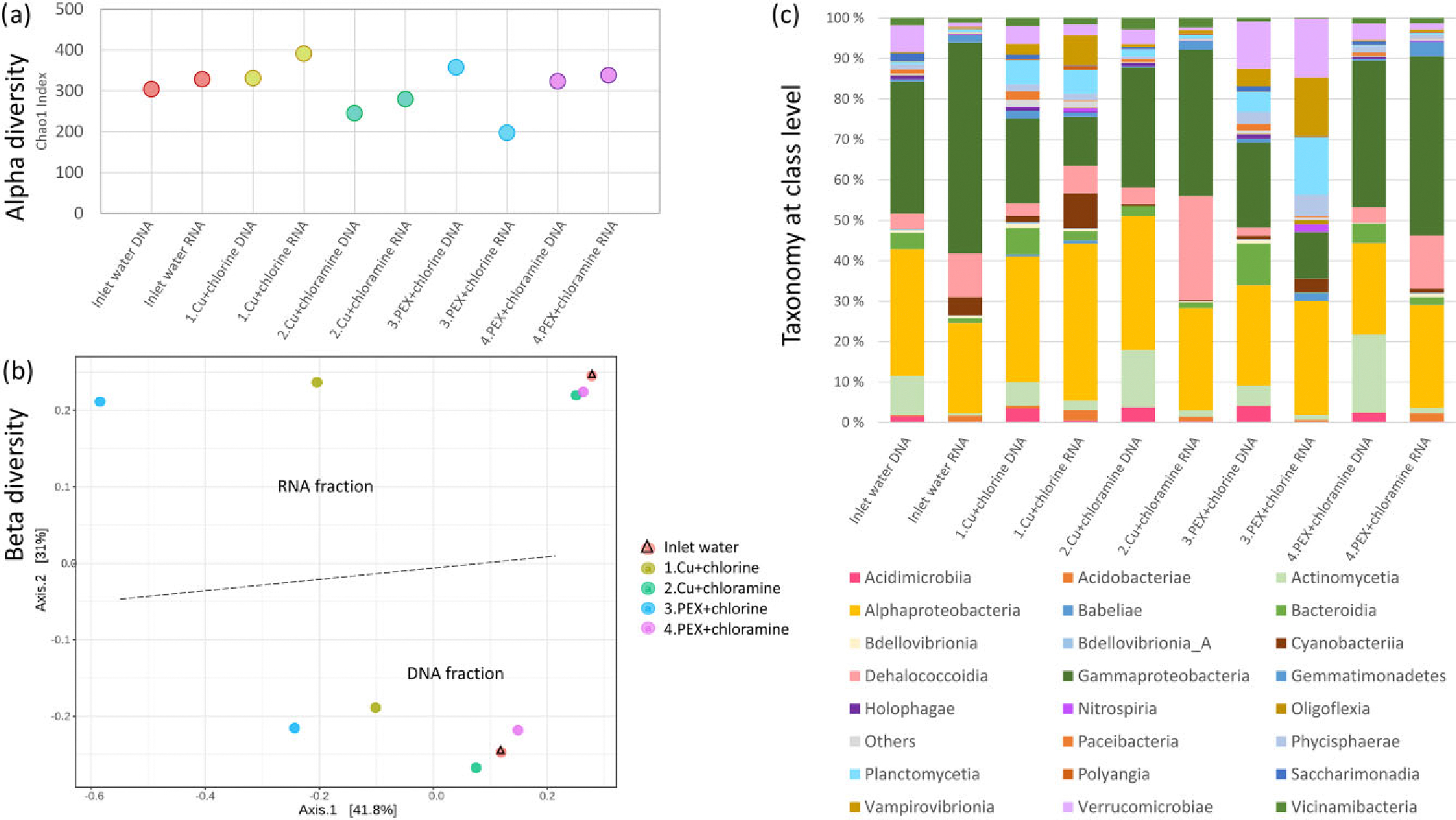
Alpha diversity by Chao1 index (a), beta diversity in principal coordinate analysis (PCoA) plot by Bray–Curtis dissimilarity index (b), and taxonomy at class level (c) of total DNA and active RNA fraction of large-volume water samples in inlet water and four study lines 1–4. The group “Others” contains classes with sequence count <200. Cu = copper pipe and PEX = cross-linked polyethylene pipe.

In large-volume water samples, taken at the last study week, alpha diversity of active fraction was higher than that of total fraction in inlet water and in copper pipelines but lower than total fraction in the chlorinated PEX pipeline and at same level in the chloraminated PEX pipeline (Fig. 5a). The dissimilarity of community composition between active and total fractions was observed based on Bray–Curtis dissimilarity index (*R*^2^: 0.26, *P* = 0.001, *n* = 10, PERMANOVA). Chloraminated water samples were close to inlet water on principal coordinate analysis plot, whereas chlorinated samples appeared separately (Fig. 5b). Bacterial community compositions of water samples of the chlorinated PEX pipeline were the most dissimilar compared to the samples of inlet water in both active and total fractions.

In large-volume water samples, the abundance of *Verrucomicrobiae*, *Vampirovibrionia*, *Planctomycetia*, *Phycisphaerae*, and *Nitrospiria* increased in chlorinated waters of copper and PEX pipes. The increase was strongest in active fraction of chlorinated PEX pipeline (Fig. 5c). *Gammaproteobacteria* and *Dehalococcoidia* decreased in chlorinated waters. In chloraminated waters, the abundance of *Dehalococcoidia* increased especially in active fraction, and the abundance of *Actinomycetia* increased especially in total fraction. *Acidobacteria* were present in all five samples in active fraction (0.5%–2.6%), unlike in total fraction (0.1%–0.7%), whereas *Acidimicrobiia* were present in all five samples in total fraction (1.6%–4.1%) but less abundant in active fraction (0.1%–0.5%). Also, *Paceibacteria* was more abundant in total fraction (0.7%–2.1%) of all study lines than in active fraction (0.0%–0.3%).

### Bacteria counts and physicochemical parameters

In water samples, HPC decreased when disinfectant was added and maintained a low count in all pipelines (Fig. S2). The lowest HPCs were in PEX pipe with chlorine disinfection. No significant changes were detected in ATP concentrations and total cell counts between pipe materials or disinfection chemicals in water; they remained low throughout the study. Copper concentrations decreased in both copper pipelines when disinfection was started from 0.5 to 0.2 mg l^−1^ (Fig. S2). In biofilm samples, HPC decreased when disinfection commenced and remained low in all other study lines, except chlorinated copper pipeline (Fig. S3). Statistically, HPCs were higher in copper pipe biofilms than in PEX pipe biofilms during the disinfection (*P* < 0.05, Kruskal–Wallis test). In contrast, total cell counts were higher in PEX pipe biofilms than in copper pipe biofilms (*P* < 0.05, Kruskal–Wallis test). Disinfection did not change the total cell counts. ATP concentrations were higher in copper pipes than in PEX pipes before disinfection (*P* < 0.05, Kruskal–Wallis test) and decreased when disinfection started, remaining low in all other study lines, except chlorinated copper pipeline, where the concentration stayed higher. Copper concentrations were higher in copper biofilms than in PEX biofilms (Fig. S3).

The water temperature was between 15°C and 22°C during the study in all four lines. Before starting the disinfection, the temperature increased by 1°C–2°C, and during the disinfection it decreased by 3°C–4°C, similarly as the temperature of inlet water (Fig. S4). Water pH stayed between 7.8 and 8.2 (Fig. S4). Total chlorine concentration of water was higher in the PEX pipe with chloramine disinfection (*P* ≤ 0.001, Kruskal–Wallis test) than in the other lines (Fig.4). Mean value of total chlorine concentration in chlorinated copper pipeline was 0.07 ± 0.02 mg l^−1^, in chloraminated copper pipeline 0.11 ± 0.04 mg l^−1^, in chlorinated PEX pipeline 0.10 ± 0.04 mg l^−1^, and in chloraminated PEX pipeline 0.31 ± 0.07 mg l^−1^. In the chloraminated copper pipeline, free ammonia concentration was 0.24 ± 0.06 mg NH_3_–N l^−1^ and in chloraminated PEX pipeline 0.18 ± 0.07 mg NH_3_–N l^−1^. Nitrite concentrations were ≤0.005 mg NO_2_–N l^−1^. Absorbance at 254 nm was lower in the chlorinated PEX pipe (*P* < 0.05, Kruskal–Wallis test) than in the other pipelines, but no significant differences occurred in absorbance at 420 nm in water between the lines. EC, turbidity, absorbance 420 nm, and iron concentrations stayed at the same level during the study period (Table S2). MAP, acetate carbon, and AOC concentrations were higher in the chlorinated PEX pipeline than other pipelines and inlet water (Table S2), based on the few samples analyzed.

### Opportunistic pathogens

**Figure 6.**
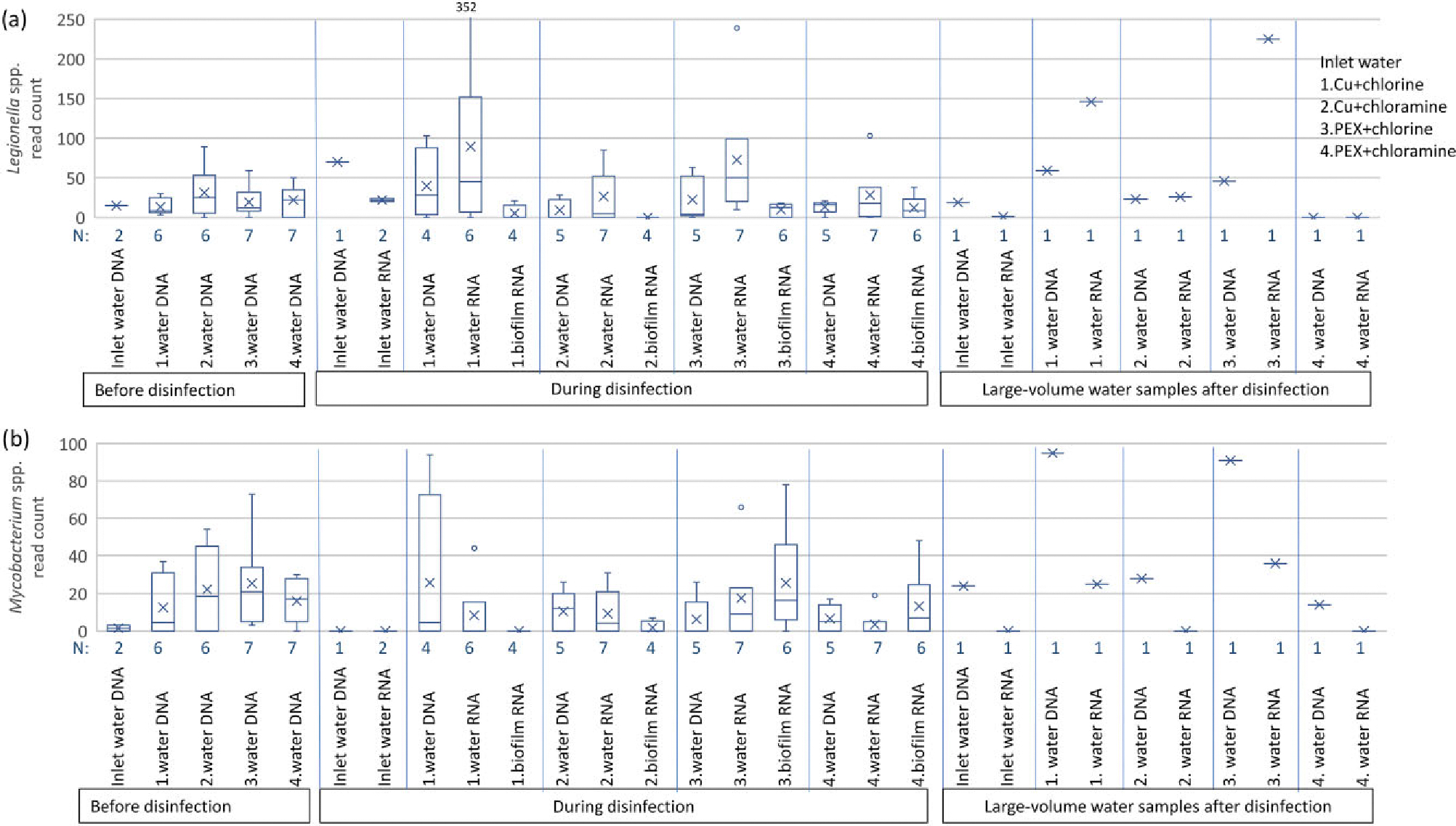
*Legionella* spp. (a) and *Mycobacterium* spp. (b) sequence read counts in total DNA and active RNA fractions of water, biofilm, and large-volume water samples. Cu = copper pipe and PEX = cross-linked polyethylene pipe. Box plot shows upper and lower quartiles, median value as line, mean value as *x*, and outliers as circles.

In all samples, a total of 97 different ASVs belonging to genus *Legionella* and 3209 *Legionella* sequence reads were detected. Only two *Legionella* ASVs were identified at species level, and they were both identified as *Legionella moravica* and were present (9 reads) in one chlorinated water sample in chlorinated PEX pipe (line 3). *Legionella* spp. read counts were detected in pipelines, even though read counts in inlet water were very low. In water samples, *Legionella* spp. read counts were higher in both total and active fractions of chlorinated pipeline waters (total: copper mean = 40, *n* = 4; PEX mean = 22, *n* = 5; active: copper mean = 89, *n* = 6, PEX mean = 73, *n* = 7) than in chloraminated pipeline waters (total: copper mean = 9, *n* = 5; PEX mean = 13, *n* = 5; active: copper mean = 26, *n* = 7, PEX mean = 28, *n* = 7), but the difference was not statistically significant (Fig. 6a). The most significant difference was in active fraction between the chlorinated PEX pipe (line 3) and the chloraminated copper pipe (line 2) (*P* = 0.06). A similar difference was seen in large-volume water samples collected at the end of the study but not observed in biofilm samples. *Legionella* spp. reads were higher in active fraction than in total fraction in chlorinated samples, indicating that *Legionella* were active in chlorinated samples.

Twelve ASVs belonging to genus *Mycobacterium* were detected from samples, but none were identified at species level. In total, 1535 sequence reads belonged to genus *Mycobacterium*. Lower *Mycobacterium* spp. read counts were detected in inlet water than in waters collected from copper and PEX pipelines even before disinfection (Fig. 6b). *Mycobacterium* spp. was higher in chlorinated copper and PEX pipes than in chloraminated copper and PEX pipes, like *Legionella* spp., but not in active fraction of copper pipes in 1 l water and biofilm samples. There, *Mycobacterium* spp. was higher in chloraminated than chlorinated water and biofilms.

In total, 7320 reads were identified as members of the genus *Pseudomonas* in all samples and were assigned to 55 ASVs with one ASV (ASV00129, species not identified) having 3345 sequence reads. At species level, *P. stutzeri* (127 reads in three samples, human opportunistic pathogen), *P. aeruginosa* (18 reads in one sample from chloraminated copper pipe (line 2, human opportunistic pathogen), *P. viridiflava* (plant pathogen), and *P. qingdaonensis* were identified. The abundances of *Pseudomonas* in the four study lines were opposite when comparing water and biofilm samples. In water, *Pseudomonas* spp. was most abundant in the chloraminated PEX pipe (line 4) (DNA mean = 301, *n* = 5; RNA mean = 49, *n* = 7), but in biofilms, in chlorinated copper pipe (line 1) (RNA mean = 558, *n* = 4). Some individual samples had a high read count of *Pseudomonas,* and in large-volume water samples no *Pseudomonas* reads were detected. The excluded ASV 08378 of contamination from the nucleic acid extraction step belonged to genus *Pseudomonas*.

## Discussion

### Combined effects of pipe material and disinfectant on DWDS bacterial communities

Disinfection, as presumed, affected the diversity of bacterial communities in water and biofilms. The dominant bacteria groups were *Alpha* - and *Gammaproteobacteria*, *Dehalococcoidia*, *Actinomycetia*, *Bacteroidia*, *Cyanobacteria*, and *Planctomycetia* like in other drinking water and biofilm communities described previously (Lu et al. 2014, Ji et al. 2015, Dias et al. 2019). However, *Actinomycetia* has been reported to be even more dominant in chlorine disinfected and PEX pipe biofilms elsewhere (Li et al. 2020, Zhang et al. 2022). Disinfection seems to be a stronger factor than pipe material in affecting active (RNA) fraction bacterial community composition of water samples, as water samples were clustered more strongly based on disinfection chemical than pipe material. Bacterial communities in water in chlorinated pipelines, even more in the chlorinated PEX pipeline, had changed the most compared with inlet water, i.e. water before the disinfection point, whereas bacterial communities in chloraminated waters were more like those in inlet water. This indicates that chlorine changed the community structure more than chloramine, even though chloramine concentration was highest in the chloraminated PEX pipe. Chlorine is a more efficient disinfection chemical and oxidant than chloramine, but it is not as stable (Copeland and Lytle 2014, Kim et al. 2024), which could contribute to the bigger change in community structure. Although disinfectants caused more difference in water samples than pipe material, there nevertheless was a difference between pipe materials with the same disinfection chemical. Water utility and disinfection type have been shown to have a greater impact than pipe material on the water microbiome in building plumbing systems (Ji et al. 2015). Copper concentrations of water in copper pipes but not in biofilms were decreased at the week when disinfection was started and stayed at lower level than before disinfection. Chlorine compounds are oxidants and can cause corrosion of copper, but formation of precipitated copper oxide layer protects from further oxidation of copper (Lytle and Liggett 2016). This may cause that copper concentration in water is decreased as disinfection is started and in biofilms it stays at the same level or accumulates from copper pipes through water flow. Decrease in copper concentrations after start of disinfection have been observed also earlier (Lehtola et al. 2004). Whether the disinfection caused the decrease here and how the decrease of copper concentration alone affected the bacterial communities were not examined in the study as control copper pipes without disinfection were not included in the experiment.

In disinfected biofilms, pipe material affected the community structure as observed earlier (Tang et al. 2021, Zhang et al. 2022). Here, alpha diversity of bacterial species, as Chao1 index, was highest in the biofilms of PEX pipe with chloramine disinfection, even though the total chlorine concentration was highest in this pipeline. Also, Li et al. (2020) observed higher species richness, as Chao1 index, for chloraminated than chlorinated high-density polyethylene pipe biofilms, in contrast to other pipe materials. Pipe material in disinfected DWDSs may impact the bacterial communities either directly through antimicrobial properties or as a nutrient source affecting the activity level of bacteria or indirectly through disinfectant demand and affecting interactions between microbes (Cullom et al. 2020). Copper has antimicrobial properties and is known to control bacteria growth in biofilms (Gomes et al. 2019) compared with plastic (Lehtola et al. 2004), and therefore, probably caused differences in biofilm composition with both chlorine and chloramine compounds also in this study. Also, copper concentration, among pH, temperature, and bacteria groups, was the most important parameter to predict changes in bacteria composition in a prediction model study with the same system and samples (Brester et al. 2020).

In total bacterial fraction (DNA) of water samples, bacterial community compositions in all four study lines were affected the most by the bacterial composition changes in inlet water and not with disinfection chemical or pipe material, as seen in beta diversity analysis. Water quality of the inlet water from the pilot-scale water treatment plant did not stay constant throughout the study and therefore affected water quality additionally to effects of disinfection and pipe material. Also, Inkinen et al. (2018) observed a weaker impact on DNA-based total bacterial communities than the clearer impact on RNA-based active bacterial communities when disinfection concentration was changed in a pilot-scale DWDS. Active and viable but dormant bacteria shown in RNA fraction are important to investigate since not all effects are seen in total DNA fraction, as also noted by Li et al. (2017) and Inkinen et al. (2018).

### Disinfection chemical efficiency in copper and PEX pipes

Total chlorine concentration measured in water was highest in the PEX pipe with chloramine disinfection. Corroding copper enhances the disinfectant decay in pipes, and new copper pipes before aging decay chlorine even more (Lehtola et al. 2005, Fu et al. 2009, Lytle and Liggett 2016, Ding et al. 2019). This may explain the significantly lower chlorine concentration in chloraminated copper pipe than chloraminated PEX pipe and slightly lower chlorine concentration in chlorinated copper pipe than chlorinated PEX pipe. Pipe material has been shown to affect chlorine concentrations in DWDSs, as PEX pipe had higher concentrations than copper in the study of Tolofari et al. (2020). Copper pipes likely degraded chlorine also in our study. Chloramine, as a disinfectant, is a more stable compound than chlorine (Lytle et al. 2021, LeChevallier et al. 2024) and probably did not react with pipe material as much as chlorine. On the other hand, chloramine is not as efficient as a disinfectant and oxidant as hypochlorite (Copeland and Lytle 2014, Kim et al. 2024), which may explain the high alpha diversity of PEX pipe biofilms with chloramine disinfection. Ammonia from chloramine disinfection may increase the amount of free ammonia and incomplete nitrification amount of nitrite if disinfection and DWDS are not operated well (Hossain et al. 2022). Ammonia may increase microbial activity and nitrite is harmful for human health. In this study ammonia and nitrite concentration were below required maximum level of 0.50 mg l^−1^ for both (European Union 2020).

Compared with this study with a residual chlorine concentration of <0.4 mg l^−1^, higher residual chlorine concentrations of 1–3.8 mg l^−1^ have been investigated more often (Norton et al. 2004, Tolofari et al. 2020, Lytle et al. 2021), although <1 mg l^−1^ residual concentrations have been evaluated as well (Ji et al. 2015, Inkinen et al. 2018, Tolofari et al. 2020). The lower concentrations are relevant in Finland and some other European countries, where disinfection is not always employed when groundwater is used as source water (Waak et al. 2019, Siponen et al. 2024).

### Effect of pipe material and disinfection on bacteria counts and ATP

Commencement of the disinfection decreased HPCs in water in all lines, except the copper line with chlorine disinfection (with the lowest measured chlorine concentration). Total cell counts were higher in plastic pipe biofilms than in copper pipe biofilms, like the report of Lehtola et al. (2004). In non-disinfected systems, HPCs and ATP were found to be lower in copper pipes than in plastic pipes (Lehtola et al. 2004), but here in the disinfected system HPCs were higher in copper pipe biofilms than in PEX pipes, and ATP was higher in chlorinated copper pipe biofilms than in chloraminated copper pipe and PEX pipes. Cell counts are logically higher in PEX pipes since PEX is not a biocide, unlike copper (Gomes et al. 2019). Also, bacteria surviving on copper pipe biofilms may be cultivable, therefore being detected as abundant HPCs, although the diversity of species is lower than in PEX pipes.

### Effects of pipe material and disinfectant on opportunistic pathogens

We observed opportunistic pathogens in pipeline waters and biofilms even though the abundances in inlet water were very low. Opportunistic pathogens may not be detected at the beginning of the distribution system but may grow and become more abundant later in the system (Lytle et al. 2021, LeChevallier et al. 2024). This may cause a health risk if pathogen abundances rise excessively and the water is consumed (Ashbolt et al. 2015, Falkinham 2020). *Legionella* spp. and *Mycobacterium* spp. were more abundant in chlorinated waters than in chloraminated water in most of the sample groups. In contrast, Buse et al. (2019) noted lower planktonic *L. pneumophila* counts in chlorinated water than in chloraminated water. However, chloramination has also earlier shown advantages in controlling *Legionella* spp. (Xi et al. 2024), and in biofilms Buse et al. (2019) also reported that chloramination controlled *L. pneumophila* more effectively on copper pipes. An exception to more effective control of opportunistic pathogens by chloramination over chlorination in this study was in active fraction in copper pipes, where *Mycobacterium* spp. was slightly more abundant in chloraminated than chlorinated water biofilms. The result supports the earlier observation of lower *Mycobacterium* abundance on chlorinated (residue of 2–3 mg l^−1^) copper pipes than on chloraminated copper pipes by Norton et al. (2004).

When comparing the effect of pipe material on opportunistic pathogens in biofilms, *Legionella* spp. and *Mycobacterium* spp. were slightly but not significantly lower in active fraction in copper pipes than in PEX pipes during the disinfection. Copper pipes have been observed to decrease *Legionella* and *Mycobacterium* occurrence (Proctor et al. 2017, Inkinen et al. 2018, Buse et al. 2019), however, not in all studies (Norton et al. 2004, Lu et al. 2014).

While chlorination in the PEX pipeline caused the biggest shift in composition of active bacterial communities in RNA water samples, *Legionella* and *Mycobacterium* were mainly more abundant in chlorinated than chloraminated waters. In biofilms, alpha diversity was lower, changes in bacteria classes relative to inlet water were higher, and abundance of *Legionella* spp. and *Mycobacterium* was slightly lower in copper pipes. The results indicate that when controlling health risk, the effects on both water and biofilm must be included in the evaluation of pipe material and disinfection chemical in DWDSs.

## Supplementary Material

Supplementary Material

## Figures and Tables

**Table 1.** Number of samples Included In bacterial community analysis and mean and standard deviation of their sequence reads. na = not applicable.

	Water before disinfection		Water during disinfection		Water during disinfection		Biofilms during disinfection		Large-volume water samples after disinfection			
	DNA		DNA		RNA		RNA		DNA		RNA	
	*N*	reads	*N*	reads	*N*	reads	*N*	reads	*N*	reads	*N*	reads

Inlet water	2	2800 ± 160	1	6500	2	28 000 ± 19 000	na	na	1	37 000	1	54 000
Line 1. Copper pipe, chlorine	6	9000 ± 8300	4	3800 ± 3800	6	15 000 ± 17 000	4	3500 ± 1800	1	14 000	1	37 000
Line 2. Copper pipe, chloramine	6	9300 ± 7900	5	2400 ± 2300	7	27 000 ± 30 000	4	1900 ± 870	1	13 000	1	22 000
Line 3. PEX pipe, chlorine	7	11 000 ± 8 900	5	1600 ± 700	7	9100 ± 7300	6	18 000 ± 12 000	1	27 000	1	61 000
Line 4. PEX pipe, chloramine	7	6200 ± 4200	5	7600 ± 9700	7	23 000 ± 25 000	6	6000 ± 2200	1	35 000	1	30 000

## Data Availability

The gene sequences generated for this study were submitted to the Short Read Archive (SRA Archive). BioSample metadata are publicly available in the NCBI database (https://www.ncbi.nlm.nih.gov/sra) under BioProject accession number PR-JNA509718.

## Impact Statement

It is well known that disinfectant residual and pipe material influence the composition and diversity of the bacterial community in the drinking water systems. Analysis of the combined effects of these factors on the community composition of active and dormant bacteria is required to understand the function of this ecosystem.
