# Supplementary material for: Effect of pipe material and disinfectant on active bacterial communities in drinking water and biofilms
Source: J Appl Microbiol. Author manuscript; Available in PMC 2025 Jul 6. (PMC12228834; doi:10.1093/jambio/lxaf004)
Supplement: Supplementary Material [file NIHMS2066845-supplement-Supplementary_Material.pdf]

Supplementary Data

EFFECT OF PIPE MATERIAL AND DISINFECTANT ON ACTIVE BACTERIAL COMMUNITIES IN DRINKING WATER AND BIOFILMS

Sallamaari Siponen[a,b,*], Jenni Ikonen[b], Vicente Gomez-Alvarez[c], Anna-Maria Hokajärvi[b], Matti Ruokolainen[a,1], Balamuralikrishna Jayaprakash[b], Mikko Kolehmainen[a], Ilkka T. Miettinen[b,d], Tarja Pitkänen[b,e], Eila Torvinen[a]

[a]*University of Eastern Finland, Department of Environmental and Biological Sciences, P.O. Box 1627, FI-70211 Kuopio, Finland.*

[b]*Finnish Institute for Health and Welfare, Department of Public Health, P.O. Box 95, FI-70701 Kuopio, Finland.*

[c]*U.S. Environmental Protection Agency, Office of Research and Development, 26W. Martin Luther King Dr., Cincinnati, OH 45268, United States of America.*

[d] *Aalto University, Water and Environmental Engineering, Tietotie 1E, FI-02150, Espoo, Finland*

[e]*University of Helsinki, Faculty of Veterinary Medicine, Department Food Hygiene and Environmental Health, P.O. Box 66, FI-00014 Helsinki, Finland.*

[1] Present address: City of Kuopio, Environmental Health Services, P.O. Box 228, FI-70101 Kuopio, Finland

*Corresponding Author

*University of Eastern Finland, Department of Environmental and Biological Sciences, P.O. Box 1627, 70211 Kuopio, Finland

E-mail address: sasipone@uef.fi

Table S1. Weekly sampling. Dark green color represents weeks when samples for bacterial community, total cell count, and adenosine triphosphate analyses were collected. + = Week when heterotrophic plate count and physico-chemical analyses were conducted, LV = Weeks when large-volume water samples from inlet water and waters from four pipelines were collected.

Sampling group	No disinfection									Disinfection going on									
	No sampling	Sampling weeks																	
		1	2	3	4	5	6	7a	7b	8	9	10	11	12	13	14	15	16	17
Inlet water	Pipeline biofilms growing for 20 days before the first sampling	+	+	+	+	+	+	+	+	+	+	+	+	+	+	+	+	+	+ LV
Waters of four pipelines		+	+	+	+	+	+	+	+	+	+	+	+	+	+	+	+	+	+ LV
Biofilms of four pipelines		+	+	+	+	+	+	+	+	+	+	+	+	+	+	+	+	+	+
Month	June			July				August						September				October	

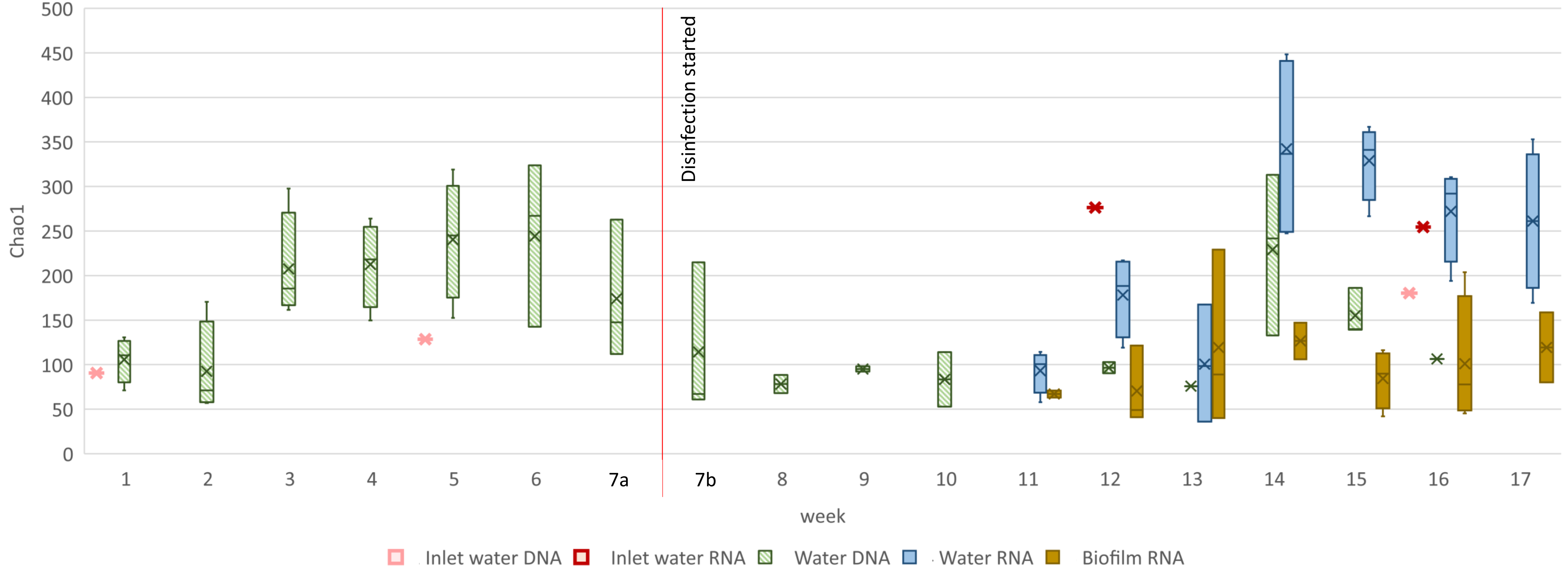

Figure S1. Alpha diversity by Chao1 index of DNA fraction of water samples at the sampling weeks 1-17 (results from weeks 11 and 17 are missing due to sequence count below 1009 in the samples) and RNA fraction of water and biofilm samples at the sampling weeks 11-17. All four study lines are grouped together.

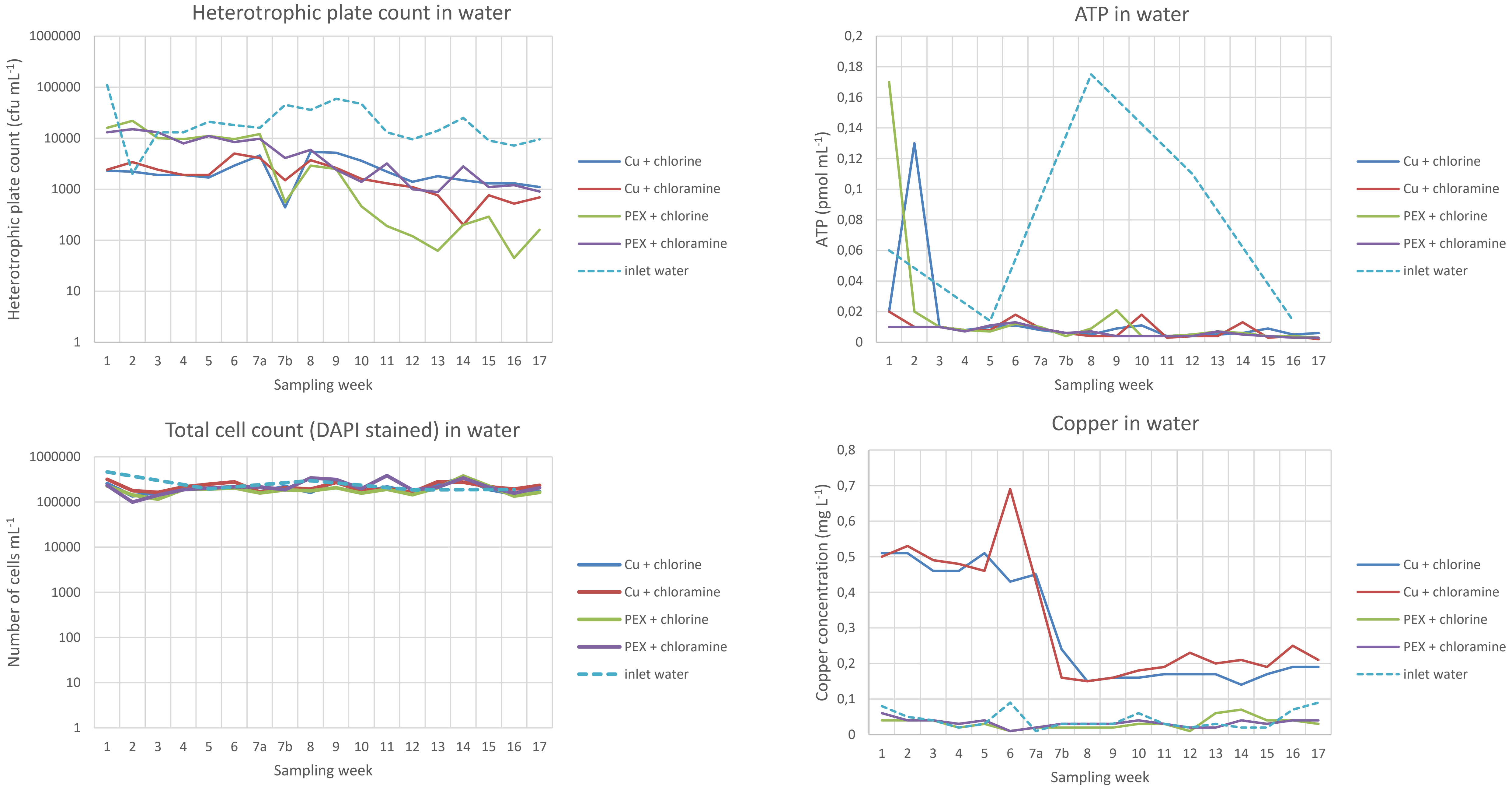

Figure S2. Heterotrophic plate count, adenosine triphosphate (ATP) concentrations, total cell counts stained with DAPI (4.6-diamidino-2-phenylindole dihydrochloride), and copper concentrations in water samples before disinfection (weeks 1 to 7a) and during disinfection (weeks 7b to 17).

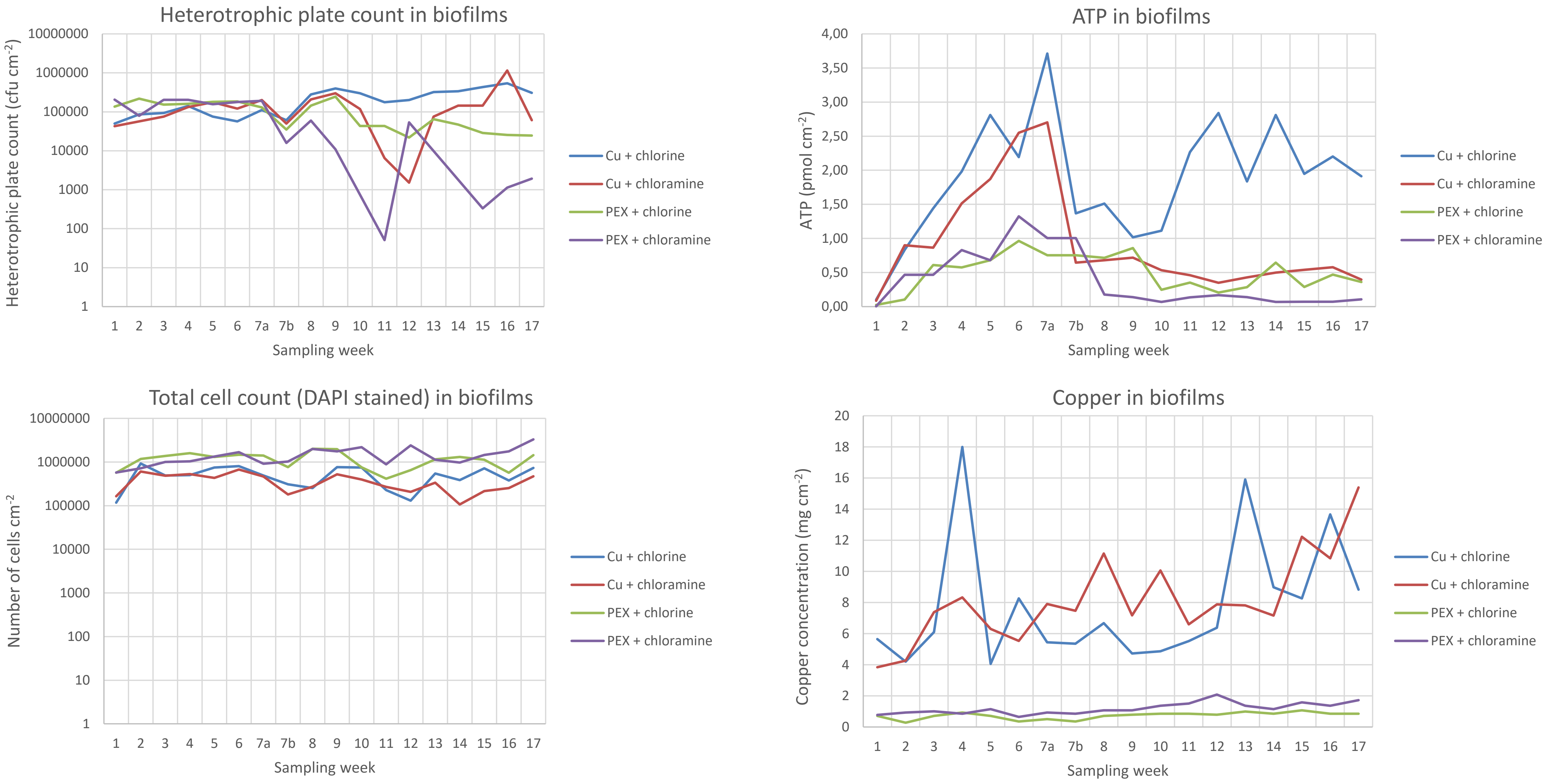

Figure S3. Heterotrophic plate count, adenosine triphosphate concentrations (ATP), total cell counts stained with DAPI (4.6-diamidino-2-phenylindole dihydrochloride), and copper concentrations in biofilm samples before disinfection (weeks 1 to 7a) and during disinfection (weeks 7b to 17).

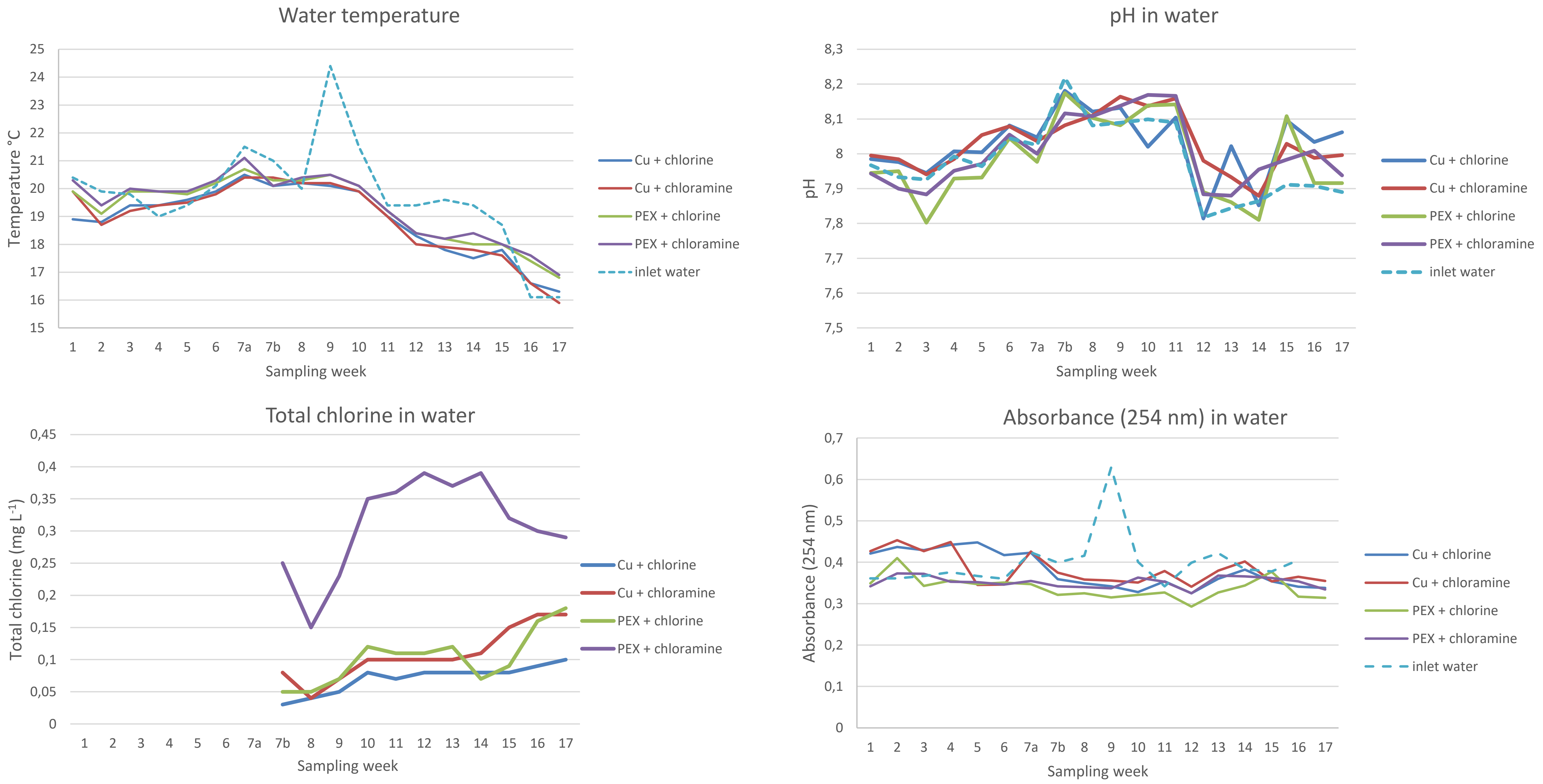

Figure S4. Temperature, total chlorine, pH, and absorbance of wavelength of 254 nm in water during the sampling period. Total chlorine concentrations were measure after the sampling began at the week 7 (second sampling 7b).

Table S2. Electric conductivity (EC), turbidity, absorbance at wavelength 420 nm, iron, microbially available phosphorus (MAP), acetate carbon (acetate-C), and assimilable organic carbon (AOC) in inlet water, pipeline waters and biofilms before and during the disinfection time period. Unit of iron concentration is mg L^{-1} for water samples and mg cm^{-2} for biofilm samples. N=number of samples, na=not applicable.

	**Sample group**	**N**	**EC (µS cm^{-1})**	**Turbidity (NTU)**	**Absorbance 420 nm**	**Iron, Fe**	**N**	**MAP (µg-PO_4-P L^{-1})**	**N**	**Acetate-C (µg L^{-1})**	**Total AOC (µg L^{-1})**
Inlet water	Inlet water before disinfection time period	7	209±6	0.48±0.56	0.04±0.03	0.25±0.27	2	0.19±0.09	5	49±7	104±12
	Inlet water during disinfection time period	11	225±13	0.88±0.93	0.03±0.04	0.29±0.20	5	<0.08	5	74±63	117±66
1. Cu + chlorine	Water before disinfection	7	207±6	0.21±0.02	0.02±0.01	0.08±0.01	3	<0.08	na	na	na
	Water during disinfection	11	224±13	0.32±0.49	0.02±0.01	0.10±0.02	2	0.23±0.19	2	52±22	115±22
	Biofilms during disinfection	11	na	na	na	3.75±1.59	na	na	na	na	na
2. Cu + chloramine	Water before disinfection	7	208±6	0.34±0.37	0.02±0.01	0.11±0.07	3	<0.08	na	na	na
	Water during disinfection	11	225±14	0.34±0.49	0.02±0.01	0.10±0.02	2	0.12±0.08	2	61±16	150±20
	Biofilms during disinfection	11	na	na	na	4.26±0.79	na	na	na	na	na
3. PEX + chlorine	Water before disinfection	7	209±7	0.20±0.12	0.02±0.01	0.11±0.06	2	<0.08	3	46±8	121±18
	Water during disinfection	11	225±14	0.27±0.39	0.02±0.01	0.09±0.02	2	0.70±0.67	2	108±43	178±38
	Biofilms during disinfection	11	na	na	na	2.79±1.04	na	na	na	na	na
4. PEX + chloramine	Water before disinfection	7	209±7	0.19±0.07	0.02±0.01	0.10±0.04	2	<0.08	3	62±15	109±16
	Water during disinfection	11	225±14	0.29±0.39	0.02±0.01	0.09±0.03	2	<0.08	1	57	117
	Biofilms during disinfection	11	na	na	na	4.03±1.41	na	na	na	na	na
